# Switching immune signals on and off

**DOI:** 10.7554/eLife.07204

**Published:** 2015-04-02

**Authors:** Nam Chu, Philip A Cole

**Affiliations:** Department of Pharmacology and Molecular Sciences, Johns Hopkins University School of Medicine, Baltimore, United States; Department of Pharmacology and Molecular Sciences, Johns Hopkins University School of Medicine, Baltimore, United Statespcole@jhmi.edu

**Keywords:** B-cell signalling, tyrosine kinase, protein structure, *E. coli*

## Abstract

Bruton's tyrosine kinase, an enzyme that is important for B cell function, can be activated in a number of ways.

**Related research article** Wang Q, Vogan EM, Nocka LM, Rosen CE, Zorn JA, Harrison SC, Kuriyan J. 2015. Autoinhibition of Bruton's tyrosine kinase (Btk) and activation by soluble inositol hexakisphosphate. *eLife* **4**:e06074. doi: 10.7554/eLife.06074

**Image** The enzyme Btk has crucial roles in tyrosine kinase signaling and the development of certain immune cells

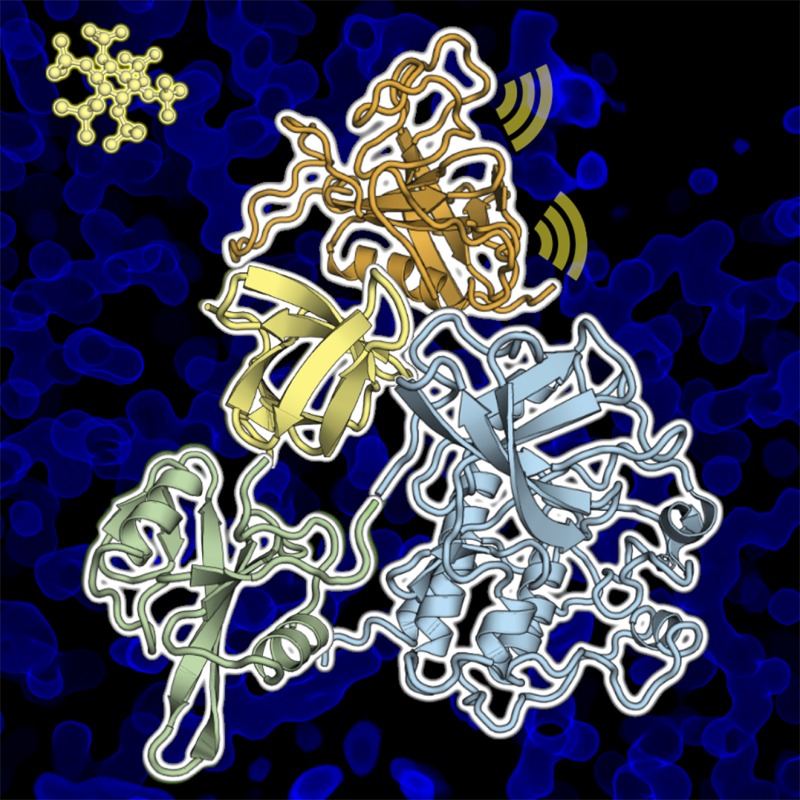

B cells are immune cells that protect the body against pathogens by making antibodies that mark the pathogens for destruction. B cells are activated by signals that are produced when enzymes called non-receptor tyrosine kinases help to phosphorylate proteins inside the B cells.

One such enzyme is Bruton's tyrosine kinase (Btk), and mutations in this enzyme are known to cause an immunodeficiency disease called X-linked agammaglobulinemia (Rawlings et al., 1993; Thomas et al., 1993). People with this disease completely lack B cells, have low levels of antibodies, and experience recurring infections (Aalipour and Advani, 2013). Hyperactive Btk also contributes to disease, causing cancerous B cells to proliferate, and this has motivated the development of drugs that target Btk. For example, the FDA-approved drug ibrutinib is an irreversible inhibitor of Btk (Honigberg et al., 2010) and is used to treat cancers such as mantle cell lymphoma and chronic lymphocytic leukemia (Aalipour and Advani, 2013).

Despite Btk's obvious biomedical importance, little was known about how its activity is regulated. Now, in *eLife*, Stephen Harrison of Harvard Medical School, John Kuriyan of the University of California, Berkeley and colleagues—including Qi Wang of Berkeley as first author—have used a series of elegant structural and biochemical approaches to identify unexpected new features of the molecular basis of Btk inhibition and activation (Wang et al., 2015).

Btk is composed of a series of different domains. The kinase domain, which catalyzes the phosphorylation of proteins, is connected via domains called SH2 and SH3 to the PH-TH domain (Figure 1). While the three-dimensional structures of the isolated Btk domains have previously been determined, it has not been clear how these domains interact with each other and how they regulate the kinase domain. It has been proposed that Btk is recruited to cellular membranes by a molecule embedded in the membrane called phosphatidyl inositol triphosphate (PIP3). This phospholipid engages with the PH-TH domain, and somehow activates the Btk kinase domain so that it phosphorylates itself and/or causes it to be activated by other tyrosine kinases (Mohamed et al., 2009).

**Figure 1.**
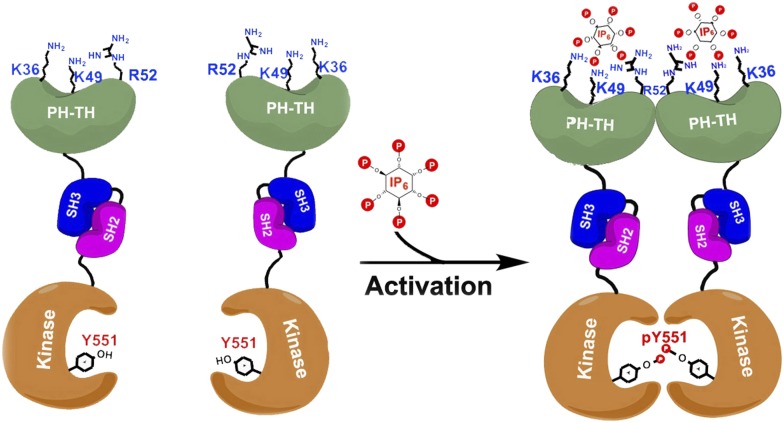
Proposed model for the activation of Bruton's tyrosine kinase (Btk) by inositol hexaphosphate (IP6). Btk consists of four domains: PH-TH (green), SH3 (blue), SH2 (purple) and the kinase domain (orange), and Wang et al. have studied how the interactions between these domains regulate the activity of the enzyme. For example, the binding of IP6 to an allosteric surface of the PH-TH domain (which contains the K36, K49 and R52 residues) can stimulate a pair of Btk molecules to form a dimer. This results in the two kinase domains phosphorylating each other at the Tyr 551 residue (Y551), which activates Btk.

Wang et al. have now solved the high-resolution crystal structures of two molecules made up of several of the Btk domains: SH3-SH2-kinase and PH-TH-kinase. These structures provide molecular details of the interactions between the Btk domains that can explain Btk autoinhibition—the ability of Btk to inhibit itself. Wang et al. found that the kinase domain is held in an inhibited state by the SH3 domain binding to the SH2-kinase linker (in a manner similar to that seen in other tyrosine kinases). Strikingly, and unique to Btk, its PH domain binds to part of the kinase called the N-lobe and cooperates with the SH2 and SH3 domains to suppress kinase activity. Wang et al. performed enzymatic experiments using vesicle-bound PIP3 to establish that Btk forms dimers as a result of the interactions between PIP3 and the PH-TH domain, and show that this dimerization stimulates the catalytic activity of Btk.

Quite by accident, Wang et al. discovered that a separate inositol phosphate molecule, a soluble molecule called inositol hexaphosphate (IP6), can bind to and activate Btk in a unique fashion. IP6 binds to a novel ‘allosteric’ surface on the PH-TH domain that is separate from the canonical PIP3-binding pocket; this binding causes pairs of Btk enzymes to form dimers, which leads to activation (Figure 1). The structural studies here are elegantly buttressed by a series of mutagenesis, enzyme kinetic, biophysical and computational analyses that lead to a compelling but complex model of multi-faceted Btk regulation.

Several provocative questions are raised by this study. Beyond IP6, can some of the pyrophosphorylated forms of phospho-inositol, such as IP7 (Chakraborty et al., 2010), also stimulate Btk? There might be a range of phospho-inositol metabolites that operate on the allosteric surface of the PH-TH domain.

Wang et al. establish that there are multiple ways of activating Btk, but are these activated species all equivalent? One can imagine that activation by PIP3-vesicle binding might lead to a different level of catalytic efficiency or substrate selectivity than activation by soluble IP6. Such different degrees of activation could promote distinct biological effects. Is there competition between vesicle-bound PIP3 and soluble IP6? Moreover, Wang et al. show that IP6 can also bind with high affinity to the canonical PH-TH/PIP3 binding cavity, which could displace Btk from membranes. Mutant forms of Btk that have been engineered to disable the two PH-TH binding surfaces should be highly informative in dissecting the roles these different activation mechanisms have in signaling networks.

What are the implications of these multiple activation states of Btk and its role in cancer? It is plausible that the varied forms of Btk activation states may show different sensitivities to kinase inhibitors, thereby influencing the clinical responses of patients treated with Btk-targeted therapeutics. Regardless of the answers to these questions, Wang et al. provide a new paradigm for thinking about tyrosine kinase signaling.
